# Increasing Quantum Correlations Based on Measurement-Induced Disturbance via a Swapping Procedure with Two-Qubit Mixed States

**DOI:** 10.3390/e23121606

**Published:** 2021-11-30

**Authors:** Chuanmei Xie, Feiyang Wu, Zhanjun Zhang, Jiawei Liang, Xiaofeng Yin

**Affiliations:** 1 School of Physics and Optoelectronics Engineering, Anhui University, Hefei 230039, China; cmxie@ahu.edu.cn (C.X.); yxfeng1357@aliyun.com (X.Y.); 2 College of Science, China University of Petroleum (Beijing), Beijing 102249, China; feiyangwoo@aliyun.com; 3 School of Information and Electronic Engineering, Zhejiang Gongshang University, Hangzhou 310018, China; 4 School of Physical Science, University of Science and Technology of China, Hefei 230026, China; ljiawei@mail.ustc.edu.cn

**Keywords:** quantum correlation swapping, measurement-induced disturbance (MID), separable two-qubit mixed state

## Abstract

In this paper, quantum correlation (QC) swapping for certain separable two-qubit mixed states is treated. A QC quantifier, measurement-induced disturbance (MID) (Luo in Phys Rev A 77:022301, 2008), is employed to characterize and quantify QCs in the relevant states. Properties of all QCs in the swapping process are revealed. Particularly, it is found that MID can be increased through QC swapping for certain separable two-qubit mixed states.

## 1. Introduction

In 2001, Ollivier and Zurek [1] exposed a surprising feature that there exist quantum correlations (QCs) in some separable states, where it is obvious that quantum entanglements do not occur. This distinct phenomenon started a new era. In this new era, people no longer believed that quantum entanglement was the avatar of QC and they were equivalent. Besides quantum entanglement, there is QC beyond entanglement (QCBE). Moreover, from then on, people gradually began to pay close attention to the new kind of QC, i.e., QCBEs. Several years later, a number of works [2,3,4,5,6,7,8,9,10,11,12,13,14,15] about QCBEs emerged, including its recognition and applications. Consequently, recently, QCBE study has formed a hot field in quantum information and computation, and many methods have been proposed or developed to investigate QCBEs in various quantum systems.

In some quantum tasks, long-distance QCs are indispensable. As for the case of long-distance entanglement, quantum entanglement repeaters are usually employed. The core technique in the repeaters is entanglement swapping [16,17,18,19,20,21,22]. Entanglement swapping can entangle a bipartite system without any previous entanglement. In addition, entanglement swapping was used as a technique to increase long-distance shared quantum entanglement [23].

Recently, quantum entanglement swapping was generalized to QC swapping [24,25,26]. In QC swapping, the relevant QCs can be quantum entanglement, QCBEs, or both of them. It is found that swapping of QCBEs can be realized in a way similar to that of entanglement swapping. However, in the existing studies about the swapping of QCBEs, QCBE in the final state cannot exceed that in the initial state. Hence, one tends to believe that although QC swapping can realize long-distance shared QCBEs, it cannot increase long-distance shared QCBE. Naturally, a problem is arising. Are there some special circumstances in which QC swapping can increase long-distance shared QCBE? The answer is positive. In this study, we will innovatively present a special case.

To be concrete, in this paper, we will consider a comparatively complicated case, where the two QCs to be swapped inhabit a pair of separable two-qubit mixed states with four host qubits distributed among three remote nodes, the swapping is realized via four Bell state measurements, and all QCs are quantified by measurement-induced disturbance [2]. The motivations in our study are fourfold: (1) To find whether quantum correlation swapping can be realized through separable two-qubit mixed states. (2) If yes, to explore the characteristics of the quantum correlation in the final states after quantum swapping. (3) To find the relationships between the quantum correlations in the final state and the ones in the initial states. In particular, to explore whether the special case can increase long-distance shared QCBE or not. (4) To study the physical origins of the above characteristics. Through concrete investigations in the following study, we will show the following essential results: (a) Quantum correlation swapping can be realized through separable two-qubit mixed states; (b) some distinct characteristics of the quantum correlation in the final states after quantum swapping can be obtained; (c) in the special QC swapping case, the long-distance shared QCBE can be realized and increased.

The rest of this paper is outlined as follows. In Section 2, the QC swapping in our case is described. In Section 3, measurement-induced disturbance is employed to characterize and quantify all QCs which occur in the swapping process. In Section 4, some analyses, discussions, and comparisons about the QCs are given. Finally, a concise summary is given in Section 5.

## 2. Quantum Correlation Swapping between Two Separable Two-Qubit Mixed States

In this paper, we will consider the separable two-qubit mixed states as the initial states for QC swapping. The separable two-qubit mixed states are taken as the following forms [27]:

(1) $$\begin{array}{cc} & \rho_{ab}\left(q_{1}\right)=q_{1}{|00\rangle }_{ab}\langle 00|+\left(1-q_{1}\right){|1+\rangle }_{ab}\langle 1+|, \end{array}$$

(2) $$\begin{array}{cc} & \rho_{cd}\left(q_{2}\right)=q_{2}{|00\rangle }_{cd}\langle 00|+\left(1-q_{2}\right){|1+\rangle }_{cd}\langle 1+|, \end{array}$$

where $q_{1}$ and $q_{2}$ are real, $q_{1},q_{2}\in \left(0,1\right)$ and $|+\rangle =(|0\rangle +|1\rangle )/\sqrt{2}$. Incidentally, in this paper, $q_{1}=q_{2}=0$ and $q_{1}=q_{2}=1$ are excluded because the corresponding states are trivial product ones, which are unhelpful and meaningless for our present study of QC swapping. It is worth mentioning that the two initial two-qubit mixed states are separable, due to the states consisting of arbitrary mixtures of two bi-qubit product pure states. That is to say, in these two initial states, there is no entanglement in them. One can also easily prove the no-entanglement property by calculating the zero entanglements [28] in them.

To realize the QC swapping, the middle bipartite measurements are respectively selected as the four Bell states, i.e.,

(3) $$\begin{array}{cc} & {|\Phi \rangle }_{ac}^{\pm }=(|00\rangle \pm \left|11\rangle \right)/\sqrt{2}, \end{array}$$

and

(4) $$\begin{array}{cc} & {|\Psi \rangle }_{ac}^{\pm }=(|01\rangle \pm \left|10\rangle \right)/\sqrt{2}. \end{array}$$

Then, after the middle measurement, the initial states $\rho_{ab}⊗\rho_{cd}$ collapse to the final state $\rho_{bd}$, i.e.,

(5) $$\begin{array}{cc} & \rho_{bd}{=}_{ac}\langle \phi |\rho^{ab}⊗\rho^{cd}{|\phi \rangle }_{ac}{/tr[}_{ac}\langle \phi |\rho^{ab}⊗\rho^{cd}{|\phi \rangle }_{ac}], \end{array}$$

where the middle measurement ${|\phi \rangle }_{ac}$s are selected as ${|\Phi \rangle }_{ac}^{\pm }$ and ${|\Psi \rangle }_{ac}^{\pm }$, respectively.

Substituting Equations (1)–(4) into Equation (5) and for the measurements ${|\Phi \rangle }_{ac}^{\pm }$, after some deductions, one can obtain

(6) $$\begin{array}{cc} & \rho_{bd}^{1}\left(q_{1},q_{2}\right)=\left(\begin{array}{cccc} \alpha_{1} & \beta_{1} & \beta_{1} & \beta_{1}\\ \beta_{1} & \beta_{1} & \beta_{1} & \beta_{1}\\ \beta_{1} & \beta_{1} & \beta_{1} & \beta_{1}\\ \beta_{1} & \beta_{1} & \beta_{1} & \beta_{1} \end{array}\right),\;\; \end{array}$$

where $\alpha_{1}=\frac{4q_{1}q_{2}+\left(1-q_{1}\right)\left(1-q_{2}\right)}{4q_{1}q_{2}+4\left(1-q_{1}\right)\left(1-q_{2}\right)}$, $\beta_{1}=\frac{\left(1-q_{1}\right)\left(1-q_{2}\right)}{4q_{1}q_{2}+4\left(1-q_{1}\right)\left(1-q_{2}\right)}$.

As for the measurement ${|\Psi \rangle }_{ac}^{\pm }$, after some derivations, another final state can be obtained, i.e.,

(7) $$\begin{array}{cc} & \rho_{bd}^{2}\left(q_{1},q_{2}\right)=\left(\begin{array}{cccc} \frac{1}{2} & \alpha_{2} & \beta_{2} & 0\\ \alpha_{2} & \alpha_{2} & 0 & 0\\ \beta_{2} & 0 & \beta_{2} & 0\\ 0 & 0 & 0 & 0 \end{array}\right),\;\; \end{array}$$

where $\alpha_{2}=\frac{q_{1}\left(1-q_{2}\right)}{2q_{1}\left(1-q_{2}\right)+2q_{2}\left(1-q_{1}\right)}$, $\beta_{2}=\frac{q_{2}\left(1-q_{1}\right)}{2q_{1}\left(1-q_{2}\right)+2q_{2}\left(1-q_{1}\right)}$.

Obviously, for the middle Bell state measurements ${|\Phi \rangle }_{ac}^{+}$ and ${|\Phi \rangle }_{ac}^{-}$, the two final states obtained through QC swapping are equivalent, denoted as $\rho_{bd}^{1}\left(q_{1},q_{2}\right)$. As for ${|\Psi \rangle }_{ac}^{+}$ and ${|\Psi \rangle }_{ac}^{-}$, the two corresponding final states are also the same, represented by $\rho_{bd}^{2}\left(q_{1},q_{2}\right)$.

It should be noted that the two kinds of final states in Equations (6) and (7) remain separable. One can easily prove the separability due to the entanglement calculations [28]. The separable final states tell us that in the process of QC swapping, the middle Bell state measurements do not introduce any entanglement into the final state.

## 3. Measurement-Induced Disturbance in the Initial States and Final States

Recently, a QC measure named measurement-induced disturbance (MID) has been attracting considerable attention for its easy computability. It was originally put forward by Luo [2]. It is defined as the difference between the total correlation quantified by quantum mutual information of the relevant state and its special classical correlation. The special classical correlation in a state is determined by measuring both subsystems with the eigenvectors of marginal states as the measuring bases.

In this section, we will use the QC quantifier, i.e., MID, to quantify the QCs in the relevant states in the QC swapping, i.e., initial states and final states.

### 3.1. MIDs in the Initial States $\rho_{ab}\left(q_{1}\right)$ and $\rho_{cd}\left(q_{2}\right)$

For the two initial states $\rho_{ab}\left(q_{1}\right)$ and $\rho_{cd}\left(q_{2}\right)$, MIDs can be expressed as follows [27]:

(8) $$\begin{array}{cc} Q(\rho_{ab})\; & =-P_{00}^{ab}\log_{2}P_{00}^{ab}-P_{01}^{ab}\log_{2}P_{01}^{ab}-P_{10}^{ab}\log_{2}P_{10}^{ab}-P_{11}^{ab}\log_{2}P_{11}^{ab}\\ \; & \;+q_{1}\log_{2}\left(q_{1}\right)+\left(1-q_{1}\right)\log_{2}\left(1-q_{1}\right), \end{array}$$

(9) $$\begin{array}{cc} Q(\rho_{cd})\; & =-P_{00}^{cd}\log_{2}P_{00}^{cd}-P_{01}^{cd}\log_{2}P_{01}^{cd}-P_{10}^{cd}\log_{2}P_{10}^{cd}-P_{11}^{cd}\log_{2}P_{11}^{cd}\\ \; & \;+q_{2}\log_{2}\left(q_{2}\right)+\left(1-q_{2}\right)\log_{2}\left(1-q_{2}\right), \end{array}$$

where

$$\begin{array}{cc} & P_{00}^{ab}=\frac{q_{1}x_{1}^{2}}{x_{1}^{2}+y_{1}^{2}},\;P_{00}^{cd}=\frac{q_{2}x_{2}^{2}}{x_{2}^{2}+d^{2}},\\ & P_{01}^{ab}=\frac{q_{1}y_{1}^{2}}{x_{1}^{2}+y_{1}^{2}},\;P_{01}^{cd}=\frac{q_{2}y_{2}^{2}}{x_{2}^{2}+d^{2}},\\ & P_{10}^{ab}=\frac{1-q_{1}}{2}\frac{{\left(x_{1}+y_{1}\right)}^{2}}{x_{1}^{2}+y_{1}^{2}},\;P_{10}^{cd}=\frac{1-q_{2}}{2}\frac{{\left(x_{2}+y_{2}\right)}^{2}}{x_{2}^{2}+y_{2}^{2}},\\ & P_{11}^{ab}=\frac{1-q_{1}}{2}\frac{{\left(x_{1}-y_{1}\right)}^{2}}{x_{1}^{2}+y_{1}^{2}},\;P_{11}^{cd}=\frac{1-q_{2}}{2}\frac{{\left(x_{2}-y_{2}\right)}^{2}}{x_{2}^{2}+y_{2}^{2}}, \end{array}$$

with $x_{1}=1-q_{1}$, $y_{1}=\sqrt{{\left(1-q_{1}\right)}^{2}+q_{1}^{2}}-q_{1}$, $x_{2}=1-q_{2}$, $y_{2}=\sqrt{{\left(1-q_{2}\right)}^{2}+q_{2}^{2}}-q_{2}$.

### 3.2. MIDs in the Final State $\rho_{bd}^{1}\left(q_{1},q_{2}\right)$

Within the framework MID, the total correlation in $\rho_{bd}^{1}$ is

(10) $$\begin{array}{cc} I(\rho_{bd}^{1}) & =S\left(\rho_{b}^{1}\right)+S\left(\rho_{d}^{1}\right)-S\left(\rho_{bd}^{1}\right), \end{array}$$

where S(·) denotes von Neumann entropy, $\rho_{b}^{1}$ and $\rho_{d}^{1}$ are marginal states of $\rho_{bd}^{1}$. The explicit forms of the marginal states are

(11) $$\begin{array}{cc} & \rho_{b}^{1}=\left(\alpha_{1}+\beta_{1}\right){|0\rangle }_{b}\langle 0|+2\beta_{1}{(|0\rangle }_{b}\langle 1|+{|1\rangle }_{b}\langle 0|+{|1\rangle }_{b}\langle 1|), \end{array}$$

(12) $$\begin{array}{cc} & \rho_{d}^{1}=\left(\alpha_{1}+\beta_{1}\right){|0\rangle }_{d}\langle 0|+2\beta_{1}{(|0\rangle }_{d}\langle 1|+{|1\rangle }_{d}\langle 0|+{|1\rangle }_{d}\langle 1|). \end{array}$$

It is easy to work out

(13) $$\begin{array}{cc} & S\left(\rho_{b}^{1}\right)=1-\left[\left(1+u_{1}\right)\log_{2}\left(1+u_{1}\right)+\left(1-u_{1}\right)\log_{2}\left(1-u_{1}\right)\right]/2, \end{array}$$

(14) $$\begin{array}{cc} & S\left(\rho_{d}^{1}\right)=1-\left[\left(1+u_{1}\right)\log_{2}\left(1+u_{1}\right)+\left(1-u_{1}\right)\log_{2}\left(1-u_{1}\right)\right]/2, \end{array}$$

(15) $$\begin{array}{cc} & S\left(\rho_{bd}^{1}\right)=1-\left[\left(1+t_{1}\right)\log_{2}\left(1+t_{1}\right)+\left(1-t_{1}\right)\log_{2}\left(1-t_{1}\right)\right]/2, \end{array}$$

where $u_{1}=\sqrt{1-8\beta_{1}\left(\alpha_{1}-\beta_{1}\right)}$ and $t_{1}=\sqrt{1-12\beta_{1}\left(\alpha_{1}-\beta_{1}\right)}$.

Now, let us turn to the classical correlation in $\rho_{bd}^{1}$. In the framework of MID, the measurements for obtaining classical correlation are selected as the product of eigenvectors of two reduced states. In this method, the spectral resolutions of the two reduced states of $\rho_{bd}^{1}$ in Equations (11) and (12) can be written as follows:

(16) $$\begin{array}{cc} & \rho_{b}^{1}=\lambda_{1,1}^{b}|N_{1,0}^{b}\rangle \langle N_{1,0}^{b}|+\lambda_{1,2}^{b}|N_{1,1}^{b}\rangle \langle N_{1,1}^{b}|, \end{array}$$

(17) $$\begin{array}{cc} & \rho_{d}^{1}=\lambda_{1,1}^{d}|M_{1,0}^{d}\rangle \langle M_{1,0}^{d}|+\lambda_{1,2}^{d}|M_{1,1}^{d}\rangle \langle M_{1,1}^{d}|, \end{array}$$

where

$$\begin{array}{cc} & \lambda_{1,1}^{b}=\lambda_{1,1}^{d}=\frac{1+u_{1}}{2},\lambda_{1,2}^{b}=\lambda_{1,2}^{d}=\frac{1-u_{1}}{2},\\ & |N_{1,0}^{b}\rangle =|M_{1,0}^{d}\rangle =\frac{-f_{1}}{\sqrt{f_{1}^{2}+g_{1}^{2}}}|0\rangle +\frac{g_{1}}{\sqrt{f_{1}^{2}+g_{1}^{2}}}|1\rangle ,\\ & |N_{1,1}^{b}\rangle =|M_{1,1}^{d}\rangle =\frac{g_{1}}{\sqrt{f_{1}^{2}+g_{1}^{2}}}|0\rangle +\frac{f_{1}}{\sqrt{f_{1}^{2}+g_{1}^{2}}}|1\rangle , \end{array}$$

with $f_{1}=4\beta_{1}$, $g_{1}=\alpha_{1}-\beta_{1}-u_{1}$.

Accordingly, the classical state of $\rho_{bd}^{1}$ can be obtained as

(18) $$\begin{array}{cc} & \chi_{\rho_{bd}^{1}}=\sum_{i,j}P_{1,ij}|N_{1,i}^{b}\rangle |M_{1,j}^{d}\rangle \langle M_{1,j}^{d}|\langle N_{1,i}^{b}|, \end{array}$$

where

(19) $$\begin{array}{cc} & P_{1,ij}=\langle M_{1,j}^{d}|\langle N_{1,i}^{b}|\rho_{bd}^{1}|N_{1,i}^{b}\rangle |M_{1,j}^{d}\rangle . \end{array}$$

Through some derivations, $P_{1,ij}$ in Equation (19) can be obtained as

(20) $$\begin{array}{cc} & P_{1,00}=\alpha_{1}F_{1}^{4}+\beta_{1}\left(6F_{1}^{2}G_{1}^{2}+G_{1}^{4}-4F_{1}G_{1}\right),\\ & P_{1,01}=P_{1,10}=\alpha_{1}F_{1}^{2}G_{1}^{2}+\beta_{1}\left(F_{1}^{4}-3F_{1}^{2}G_{1}^{2}+G_{1}^{4}\right),\\ & P_{1,11}=\alpha_{1}G_{1}^{4}+\beta_{1}\left(F_{1}^{4}+4F_{1}G_{1}+6F_{1}^{2}G_{1}^{2}\right), \end{array}$$

with $F_{1}=\frac{f_{1}}{\sqrt{f_{1}^{2}+g_{1}^{2}}}$, $G_{1}=\frac{g_{1}}{\sqrt{f_{1}^{2}+g_{1}^{2}}}$.

Accordingly, the von Neumann entropy of $\chi_{\rho_{1}^{bd}}$ can be obtained as

(21) $$S\left(\chi_{\rho_{bd}^{1}}\right)=-P_{1,00}\log_{2}P_{1,00}-P_{1,01}\log_{2}P_{1,01}-P_{1,10}\log_{2}P_{1,10}-P_{1,11}\log_{2}P_{1,11}$$

With the classical state in Equation (18), one can obtain the classical correlation in $\rho_{bd}^{1}$, i.e., the mutual information in the classical state $\chi_{\rho_{bd}^{1}}$,

(22) $$C\left(\rho_{bd}^{1}\right)=I\left(\chi_{\rho_{bd}^{1}}\right)=S\left(\chi_{\rho_{b}^{1}}\right)+S\left(\chi_{\rho_{d}^{1}}\right)-S\left(\chi_{\rho_{bd}^{1}}\right),$$

where $\chi_{\rho_{b}^{1}}$ and $\chi_{\rho_{d}^{1}}$ are marginal states of $\chi_{\rho_{bd}^{1}}$.

As a result, the quantum correlation in $\rho_{bd}^{1}$ estimated via MID can be obtained as

(23) $$Q\left(\rho_{bd}^{1}\right)=I\left(\rho_{bd}^{1}\right)-C\left(\rho_{bd}^{1}\right)=S\left(\chi_{\rho_{bd}^{1}}\right)-S\left(\rho_{bd}^{1}\right).$$

Finally, MID in the final state $\rho_{bd}^{1}$ can be written as

(24) $$\begin{array}{cc} Q(\rho_{bd}^{1})\; & =-P_{1,00}\log_{2}P_{1,00}-P_{1,01}\log_{2}P_{1,01}-P_{1,10}\log_{2}P_{1,10}-P_{1,11}\log_{2}P_{1,11}\\ \; & \;+\frac{1+t_{1}}{2}\log_{2}\left(\frac{1+t_{1}}{2}\right)+\frac{1-t_{1}}{2}\log_{2}(\frac{1-t_{1}}{2}). \end{array}$$

### 3.3. MIDs in the Final State $\rho_{bd}^{2}\left(q_{1},q_{2}\right)$

The total correlation in the final state $\rho_{bd}^{2}$ is

(25) $$I\left(\rho_{bd}^{2}\right)=S\left(\rho_{b}^{2}\right)+S\left(\rho_{d}^{2}\right)-S\left(\rho_{bd}^{2}\right)$$

where $\rho_{b}^{2}$ and $\rho_{d}^{2}$ are marginal states of $\rho_{bd}^{2}$, with

(26) $$\begin{array}{cc} & \rho_{b}^{2}=\left(\alpha_{2}+\frac{1}{2}\right){|0\rangle }_{b}\langle 0|+\beta_{2}{(|0\rangle }_{b}\langle 1|+{|1\rangle }_{b}\langle 0|+{|1\rangle }_{b}\langle 1|), \end{array}$$

(27) $$\begin{array}{cc} & \rho_{d}^{2}=\left(\beta_{2}+\frac{1}{2}\right){|0\rangle }_{d}\langle 0|+\alpha_{2}{(|0\rangle }_{d}\langle 1|+{|1\rangle }_{d}\langle 0|+{|1\rangle }_{d}\langle 1|). \end{array}$$

One can work out

(28) $$\begin{array}{cc} & S\left(\rho_{b}^{2}\right)=1-\left[\left(1+u_{2}\right)\log_{2}\left(1+u_{2}\right)+\left(1-u_{2}\right)\log_{2}\left(1-u_{2}\right)\right]/2, \end{array}$$

(29) $$\begin{array}{cc} & S\left(\rho_{d}^{2}\right)=1-\left[\left(1+u_{2}\right)\log_{2}\left(1+u_{2}\right)+\left(1-u_{2}\right)\log_{2}\left(1-u_{2}\right)\right]/2, \end{array}$$

(30) $$\begin{array}{cc} & S\left(\rho_{bd}^{2}\right)=1-\left[\left(1+t_{2}\right)\log_{2}\left(1+t_{2}\right)+\left(1-t_{2}\right)\log_{2}\left(1-t_{2}\right)\right]/2, \end{array}$$

where $u_{2}=\sqrt{1-8\alpha_{2}\beta_{2}}$ and $t_{2}=\sqrt{1-12\alpha_{2}\beta_{2}}$.

In the framework of MID, to obtain the classical state, the marginal states $\rho_{b}^{2}$ and $\rho_{d}^{2}$ in Equations (26) and (27) can be rewritten as

(31) $$\begin{array}{cc} & \rho_{b}^{2}=\lambda_{2,1}^{b}|N_{2,0}^{b}\rangle \langle N_{2,0}^{b}|+\lambda_{2,2}^{b}|N_{2,1}^{b}\rangle \langle N_{2,1}^{b}|, \end{array}$$

(32) $$\begin{array}{cc} & \rho_{d}^{2}=\lambda_{2,1}^{d}|M_{2,0}^{d}\rangle \langle M_{2,0}^{d}|+\lambda_{2,2}^{d}|M_{2,1}^{d}\rangle \langle M_{2,1}^{d}|, \end{array}$$

where

$$\begin{array}{cc} & \lambda_{2,1}^{b}=\lambda_{2,1}^{d}=\frac{1+u_{2}}{2},\\ & \lambda_{2,2}^{b}=\lambda_{2,2}^{d}=\frac{1-u_{2}}{2},\\ & |N_{2,0}^{b}\rangle =\frac{\beta_{2}}{\sqrt{\beta_{2}^{2}+f_{2}^{2}}}|0\rangle -\frac{f_{2}}{\sqrt{\beta_{2}^{2}+f_{2}^{2}}}|1\rangle ,\\ & |N_{2,1}^{b}\rangle =\frac{f_{2}}{\sqrt{\beta_{2}^{2}+f_{2}^{2}}}|0\rangle +\frac{\beta_{2}}{\sqrt{\beta_{2}^{2}+f_{2}^{2}}}|1\rangle ,\\ & |M_{2,0}^{d}\rangle =\frac{\alpha_{2}}{\sqrt{\alpha_{2}^{2}+g_{2}^{2}}}|0\rangle -\frac{g_{2}}{\sqrt{\alpha_{2}^{2}+g_{2}^{2}}}|1\rangle ,\\ & |M_{2,1}^{d}\rangle =\frac{g_{2}}{\sqrt{\alpha_{2}^{2}+g_{2}^{2}}}|0\rangle +\frac{\alpha_{2}}{\sqrt{\alpha_{2}^{2}+g_{2}^{2}}}|1\rangle , \end{array}$$

with $f_{2}=\alpha_{2}-\frac{u_{2}}{2}$ and $g_{2}=\beta_{2}-\frac{u_{2}}{2}$.

With the spectrum representation of $\rho_{bd}^{2}$, the classical state of $\rho_{bd}^{2}$ in the framework of MID can be expressed as

(33) $$\begin{array}{cc} & \chi_{\rho_{bd}^{2}}=\sum_{i,j}P_{2,ij}|N_{2,i}^{b}\rangle |M_{2,j}^{d}\rangle \langle M_{2,j}^{d}|\langle N_{2,i}^{b}|, \end{array}$$

where

(34) $$\begin{array}{cc} & P_{2,ij}=\langle M_{2,j}^{d}|\langle N_{2,i}^{b}|\rho_{bd}^{2}|N_{2,i}^{b}\rangle |M_{2,j}^{d}\rangle . \end{array}$$

After some tedious calculations, one can obtain

$$\begin{array}{cc} & P_{2,00}=\frac{1}{2}F_{2,1}^{2}F_{2,2}^{2}+\alpha_{2}F_{2,1}^{2}G_{2,2}\left(G_{2,2}-2F_{2,2}\right)+\beta_{2}F_{2,2}^{2}G_{2,1}\left(G_{2,1}-2F_{2,1}\right),\\ & P_{2,01}=\frac{1}{2}F_{2,1}^{2}G_{2,2}^{2}+\alpha_{2}F_{2,1}^{2}F_{2,2}\left(2G_{2,2}+F_{2,2}\right)+\beta_{2}G_{2,2}^{2}G_{2,1}\left(G_{2,1}-2F_{2,1}\right),\\ & P_{2,10}=\frac{1}{2}F_{2,2}^{2}G_{2,1}^{2}+\alpha_{2}G_{2,1}^{2}G_{2,2}\left(G_{2,2}-2F_{2,2}\right)+\beta_{2}F_{2,1}F_{2,2}^{2}\left(F_{2,1}+2G_{2,1}\right),\\ & P_{2,11}=\frac{1}{2}G_{2,1}^{2}G_{2,2}^{2}+\alpha_{2}G_{2,1}^{2}F_{2,2}\left(F_{2,2}+2G_{2,2}\right)+\beta_{2}F_{2,1}G_{2,2}^{2}\left(F_{2,1}+2G_{2,1}\right), \end{array}$$

with $F_{2,1}=\frac{f_{2,1}}{\sqrt{f_{2,1}^{2}+g_{2,1}^{2}}}$, $G_{2,1}=\frac{g_{2,1}}{\sqrt{f_{2,1}^{2}+g_{2,1}^{2}}}$ and $F_{2,2}=\frac{f_{2,2}}{\sqrt{f_{2,2}^{2}+g_{2,2}^{2}}}$, $G_{2,2}=\frac{g_{2,2}}{\sqrt{f_{2,2}^{2}+g_{2,2}^{2}}}$.

Accordingly, the von Neumann entropy of $\chi_{\rho_{2}^{bd}}$ can be obtained as

(35) $$S\left(\chi_{\rho_{bd}^{2}}\right)=-P_{2,00}\log_{2}P_{2,00}-P_{2,01}\log_{2}P_{2,01}-P_{2,10}\log_{2}P_{2,10}-P_{2,11}\log_{2}P_{2,11},$$

Further, the classical correlation in $\rho_{bd}^{2}$ can be obtained as

(36) $$C\left(\rho_{bd}^{2}\right)=I\left(\chi_{\rho_{bd}^{2}}\right)=S\left(\chi_{\rho_{b}^{2}}\right)+S\left(\chi_{\rho_{d}^{2}}\right)-S\left(\chi_{\rho_{bd}^{2}}\right),$$

where $\chi_{\rho_{b}^{2}}$ and $\chi_{\rho_{d}^{2}}$ are marginal states of $\chi_{\rho_{bd}^{2}}$.

Finally, the quantum correlation of $\rho_{bd}^{2}$ is consequently obtained as

(37) $$\begin{array}{cc} Q(\rho_{bd}^{2}) & =I\left(\rho_{bd}^{2}\right)-C\left(\rho_{bd}^{2}\right)\\ \; & =S\left(\chi_{\rho_{bd}^{2}}\right)-S\left(\rho_{bd}^{2}\right)\\ \; & =-P_{2,00}\log_{2}P_{2,00}-P_{2,01}\log_{2}P_{2,01}-P_{2,10}\log_{2}P_{2,10}-P_{2,11}\log_{2}P_{2,11}\\ \; & \;+\frac{1+t_{2}}{2}\log_{2}\left(\frac{1+t_{2}}{2}\right)+\frac{1-t_{2}}{2}\log_{2}(\frac{1-t_{2}}{2}). \end{array}$$

## 4. Analyses, Discussions, and Comparisons

In the last section, QCs in the initial states and final states which emerge during the swapping process are quantified by MID. In this section, we will carry out some analyses, discussions, and comparisons on them.

As mentioned above, the initial states and final states are all separable states. That is to say, there is no entanglement in any of the relevant states. Hence, in the present study, the QC swapping case is not entanglement swapping. It is a quantum correlation beyond entanglement (QCBE) swapping. The QCBE quantifier utilized in this paper is MID.

### 4.1. Features of MIDs in the Relevant States

**Figure 1:**
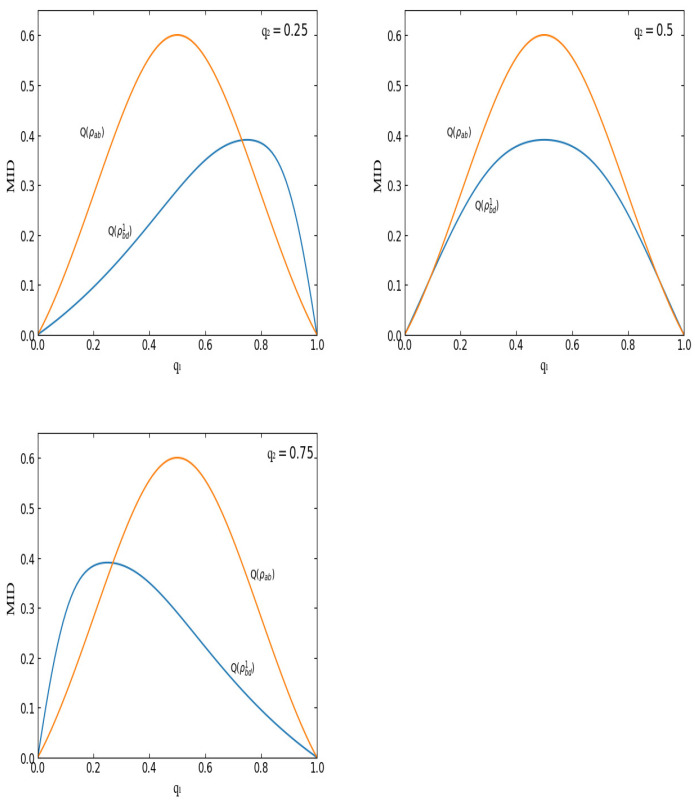
MIDs in $\rho_{ab}$ and $\rho_{bd}^{1}$ for $q_{2}=0.25,0.5,0.75,$ respectively.

Firstly, let us briefly see the monotony features of MID in the initial states for the kind of separable state ρ(q) in Equations (1) and (2). All the captured QCs increase with *q* in the region (0,1/2] and decrease with *q* in the region [1/2,1). Moreover, there exists an obvious symmetry. That is to say, QCs in both states with q=1/2±δq (0≤δq≤1/2) are the same. See Figure 1 for an example. In Figure 1, MIDs in the initial state $\rho_{ab}$ and final state $\rho_{bd}^{1}$ are described for $q_{2}=0.25,0.5,0.75$.

**Figure 2:**
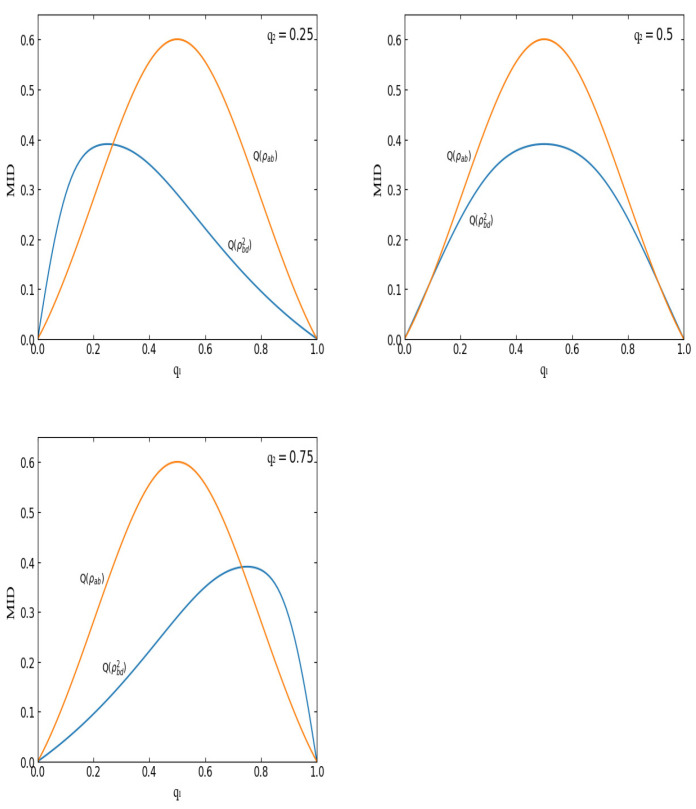
MIDs in $\rho_{ab}$ and $\rho_{bd}^{2}$ for $q_{2}=0.25,0.5,0.75,$ respectively.

Secondly, let us turn to the MIDs in the final states. In the QC swapping progress, the final states are derived from the initial states due to the middle measurements. In this paper, the middle measurement states are selected as the four Bell states, respectively. Accordingly, two kinds of final states are obtained, i.e., $\rho_{bd}^{1}$ and $\rho_{bd}^{2}$. Hence, two kinds of MIDs are derived, $Q(\rho_{bd}^{1})$ and $Q(\rho_{bd}^{2})$. Obviously, $Q(\rho_{bd}^{i})$ (i=1,2) is determined by two parameters, $q_{1}$ and $q_{2}$, which are from the initial states. In Figure 1 and Figure 2, $Q(\rho_{bd}^{1})$ and $Q(\rho_{bd}^{2})$ are depicted with $q_{1}$ for $q_{2}=0.25,0.5,0.75$, respectively.

Inspecting the QCs in the final states, i.e., $Q(\rho_{bd}^{i})$ (i=1,2) in Equations (24) and (37), one can see the following four distinct features.

**Figure 3:**
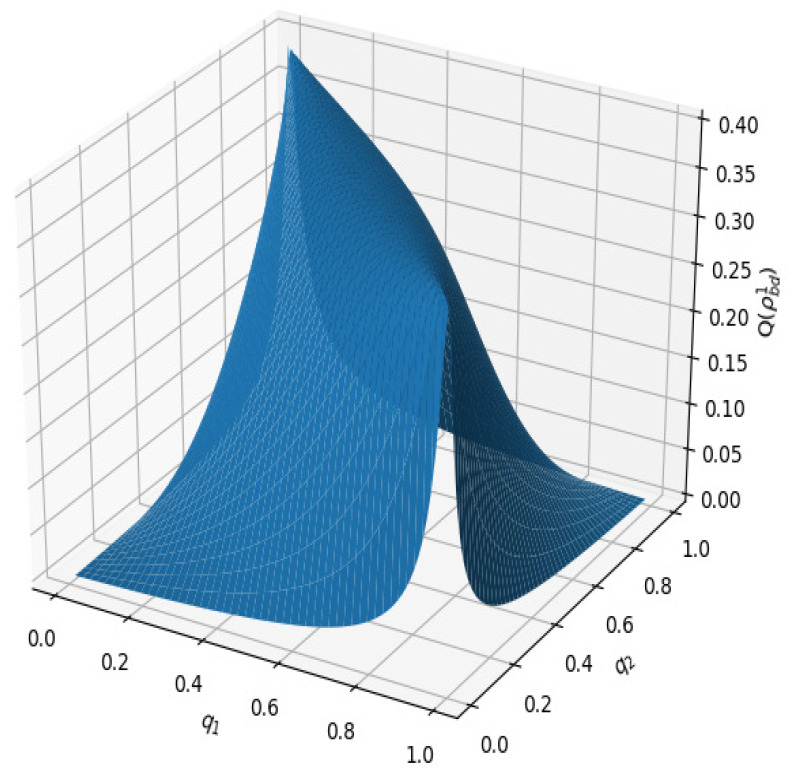
Three-dimensional image of $Q(\rho_{bd}^{1})$ with $q_{1}$ and $q_{2}$.

(i) In the region $q_{1}\in \left(0,1\right)$, $Q(\rho_{bd}^{i})$ (i=1,2) first increases, then decreases with the value of $q_{1}$, and the position of peak $Q(\rho_{bd}^{i})$ varies with the value of $q_{2}$.

(ii) The peak of $Q(\rho_{bd}^{i})$ (i=1,2) maintains a fixed value of 0.3903 for different $q_{2}$. For example, in Figure 1, the peak values of $Q(\rho_{bd}^{1})$ in the three different cases (corresponding to $q_{2}=0.25,0.5,0.75$, respectively) are all equivalent to the same value, i.e., 0.3903 (see Figure 3). In Figure 3, a three-dimensional image of $Q(\rho_{bd}^{1})$ is plotted with $q_{1}$ and $q_{2}$. Similarly, it is found that the peak of $Q(\rho_{bd}^{2})$ is also maintained at a fixed value of 0.3903.

With further inspection of the final states $\rho_{bd}^{i}$ in Equations (6) and (7), one can find that $\partial^{2}Q\left(\rho_{bd}^{1}\right)/\partial q_{1}\partial q_{2}=0$ when $q_{1}+q_{2}=1$ and $\partial^{2}Q\left(\rho_{bd}^{2}\right)/\partial q_{1}\partial q_{2}=0$ when $q_{1}=q_{2}$. That is to say, $Q(\rho_{bd}^{1})$ reaches to its maximal value at $q_{1}+q_{2}=1$ and $Q(\rho_{bd}^{2})$ reaches its maximal value at $q_{1}=q_{2}$. Moreover, in the extreme point conditions, the two final states become constant states and, correspondingly, $Q(\rho_{bd}^{i})$ are 0.3903. Hence, one can conclude that $Q(\rho_{bd}^{1})$ reaches the maximal value 0.3903 at $q_{1}+q_{2}=1$ and $Q(\rho_{bd}^{2})$ reaches the maximal value 0.3903 at $q_{1}=q_{2}$.

(iii) In the region $q_{1}\in \left(0,1\right)$, $Q(\rho_{bd}^{i})$ with $q_{2}$ and $Q(\rho_{bd}^{i})$ with $1-q_{2}$ are symmetric about $q_{1}=1/2$ (see Figure 1 for an example). Comparing the first picture ($q_{2}=0.25$) with the third one ($q_{2}=0.75$), one can find that they are symmetrical about $q_{1}=1/2$. This symmetrical feature originates from the internal symmetries in the initial states in the QC swapping process.

(iv) $Q\left(\rho_{bd}^{1}\right)\left(q_{1},q_{2}\right)=Q\left(\rho_{bd}^{2}\right)\left(q_{1},1-q_{2}\right)$. For example, the first variation diagram in Figure 1 is same with the third variation diagram in Figure 2. To be concrete, $Q\left(\rho_{bd}^{1}\right)\left(q_{1},q_{2}=0.25\right)=Q\left(\rho_{bd}^{2}\right)\left(q_{1},q_{2}=0.75\right)$.

### 4.2. QC Swapping Can Be Realized through Separable Two-Qubit Mixed States

From Figure 1 and Figure 2, one can see that QCs always exist in the initial state $\rho_{ab}$ and the final state $\rho_{bd}^{i}$ in the regions $q_{i}\in \left(0,1\right)$ (i=1,2). Obviously, another initial state $\rho_{cd}$ has the same property as $\rho_{ab}$. Hence, QC does not vanish in any of the three states. This is a distinct phenomenon. To be concrete, in the region $q_{i}\in \left(0,1\right)$,(i=1,2), QCs in the two initial states are non-zero. Correspondingly, QC in the final state is non-zero too. That is to say, QC swapping can be realized through separable two-qubit mixed states.

### 4.3. MID Can Be Increased through QC Swapping

Now, let us turn to the comparison between the MIDs before QC swapping with those after QC swapping. From Figure 1 and Figure 2, one can find a distinct feature, i.e., $Q(\rho_{bd}^{i})$ (i=1,2) can be bigger than $Q(\rho_{ab})$ in some special regions. That is to say, in our case, MID can be increased through QC swapping. Taking $Q(\rho_{bd}^{1})$ as an example, see Figure 1. For the case of $q_{2}=0.25$, in the region $q_{1}\in \left(0.731,1\right)$, values of $Q(\rho_{bd}^{1})$ are greater than those of $Q(\rho_{ab})$; for the case of $q_{2}=0.75$, in the region $q_{1}\in \left(0.269,1\right)$, values of $Q(\rho_{bd}^{1})$ are greater than those of $Q(\rho_{ab})$.

The special region for $Q(\rho_{bd}^{1})$ that is bigger than $Q(\rho_{ab})$ varies with the value of $q_{2}$. To be concrete, for different $q_{2}$, the $q_{1}$ region in which MID can be increased is different. Obviously, the special region is determined by a crosspoint of curves $Q(\rho_{ab})$ and $Q(\rho_{bd}^{1})$.

From the cases of $q_{2}=0.25$ and $q_{2}=0.75$ in Figure 1, one can find that the number of crosspoints (except for zero and one) of $Q(\rho_{ab})$ and $Q(\rho_{bd}^{1})$ is one. However, in the $q_{2}=0.5$ case, the number becomes two (see Table 1). In Table 1, the points of intersection between $Q(\rho_{ab})$ and $Q(\rho_{bd}^{i})$ are listed.

From Table 1, one can see that when $q_{2}=0.5$, the number of intersections (except for zero and one) between $Q(\rho_{ab})$ and $Q(\rho_{bd}^{i})$ is two. However, when $q_{2}\neq 0.5$, taking $q_{2}=0.25$ as an example, the number of intersections is one. Upon further inspection of the variation of the intersections with the value of $q_{2}$, one can find a phenomenon. To be concrete, when $q_{2}$ decreases from 0.5 to zero, the graph of $Q(\rho_{bd}^{1})$ moves to the right gradually. As a result, the left intersection disappears gradually. On the contrary, when $q_{2}$ increases form 0.5 to one, the graph of $Q(\rho_{bd}^{1})$ moves to the left and the right intersection disappears gradually (see Figure 1 for an example). As for the graph of $Q(\rho_{bd}^{2})$, the asymptotic behavior is adverse. That is to say, when $q_{2}$ decreases from 0.5 to zero, the graph of $Q(\rho_{bd}^{2})$ moves to the left gradually and the right intersection disappears gradually. When $q_{2}$ increases from 0.5 to one, the graph of $Q(\rho_{bd}^{2})$ moves to the right and the left intersection disappears gradually.

In a word, in some special regions, QCs in the final state can be bigger than those in the initial states. That is to say, in our considered QC swapping case, QC can be increased through the QC swapping process.

Finally, let us make some simple remarks. In this study, we consider a special case of quantum correlation swapping. The two initial states we considered are separable two-qubit mixed states. In this case, a distinct phenomenon has been found, i.e., quantum correlation can be increased through QC swapping. However, in this paper, we only consider one kind of QC quantifier, i.e., MID. This is because of its comparatively easy computability. Are the features and conclusions obtained in the study applicable for swapping QCs in other initial states via other QC measures? This is still an open question. We will pay attention to them in the near future.

## 5. Summary

To summarize, in this paper, we have considered QC swapping with separable two-qubit mixed states as the initial states. With the assistance of numerical computations, some distinct features have been exposed. In particular, it is found that MID in the final state after QC swapping can be bigger than those in the initial states before QC swapping. That is to say, MID can be increased through QC swapping.

## Figures and Tables

**Table 1:** Points of intersection between $Q(\rho_{ab})$ and $Q(\rho_{bd}^{i})$.

	[$q_{1}$,$Q(\rho_{\mathrm{bd}}^{1})$]	[$q_{1}$,$Q(\rho_{\mathrm{bd}}^{2})$]
$q_{2}=0.250$	[0.731,0.390]	[0.269,0.390]
$q_{2}=0.500$	[0.089,0.112]	[0.089,0.112]
	[0.911,0.110]	[0.911,0.110]
$q_{2}=0.750$	[0.269,0.390]	[0.731,0.390]

## Data Availability

Not applicable.
